# Improving quality of care in fragile, conflict-affected and vulnerable settings

**DOI:** 10.2471/BLT.19.246280

**Published:** 2020-01-01

**Authors:** Shamsuzzoha Babar Syed, Sheila Leatherman, Matthew Neilson, Andre Griekspoor, Dirk Horemans, Mondher Letaief, Edward Kelley

**Affiliations:** a Integrated Health Services Department, World Health Organization, avenue Appia 20, 1211 Geneva 27, Switzerland.; b Gillings School of Global Public Health, University of North Carolina, Chapel Hill, United States of America.; c Emergency Response Operations, World Health Organization, Geneva, Switzerland.; d Department of Health System Development, World Health Organization Regional Office for the Eastern Mediterranean, Cairo, Egypt.

Quality of care is central to population health. In recent years, several publications on quality of care have added to global knowledge^1^^–^^4^ and called for quality to be at the core of universal health coverage (UHC).

The term fragile, conflict-affected and vulnerable settings broadly describes situations of crisis. People living in such settings include all those experiencing humanitarian crises, prolonged disruption to critical public services, significant armed conflict, extreme adversity or acute, protracted or complex emergencies. The health service needs in such settings are significant. Estimates show that 60% of preventable maternal deaths, 53% of deaths in children younger than 5 years of age and 45% of neonatal deaths are in settings of conflict, displacement and natural disasters.^5^ Achieving UHC needs action across all countries, all settings^6^, as highlighted in the 2019 global monitoring report *Primary health care on the road to universal health coverage*.^7^

A range of constraints exist in such settings: breakdown in health systems, inadequate workforce, lack of safety and security including attacks on health care, alongside scarcity of resources.^8^ While the first priority is to restore service provision to save lives, all actors involved in provision of health services must also ensure quality of care. How can we plan for action in unstable settings?

The World Health Organization (WHO) is currently working with partners to address quality of care in fragile, conflict-affected and vulnerable settings. These efforts build on WHO’s national quality policy and strategy initiative, on an emerging academic and experiential knowledge base, and on foundational efforts on quality health services from across the humanitarian and development sectors.^9^^,^^10^ This work supports WHO’s quality improvement task team that was recently created under the global health cluster, a network of partners that works in humanitarian emergencies.

The following eight interdependent elements are proposed as key considerations in developing a strategic approach to quality action planning in such settings.^8^

First, consideration of service priorities and quality goals reflects the need to align with existing health sector priorities, as well as identifying focus conditions and populations. Second, shared local understanding of quality across the many actors in such settings provides a common language for improvement efforts across the various quality domains. Third, stakeholder mapping and engagement, building on existing coordination mechanisms, allows collaborative efforts to advance a commitment to – and capacity for – quality. Fourth, situational analysis identifies context-specific challenges to delivery of quality care. Fifth, arrangements for governance and accountability should, at a minimum, identify processes and accountability for quality, leveraging existing humanitarian coordination platforms. Sixth, action plans require a pragmatic package of quality interventions across five intervention goals: assure access and basic infrastructure; shape the system environment; reduce harm; improve clinical care; and engage patients, families and communities.^8^ Seventh, health information systems provide the health-care data required to monitor improvement across the system. In settings where routine systems may not have been established or have been disrupted, the collection of suitable health data to support delivery of quality care is needed. Finally, improvement can be monitored and measured using a pragmatic indicator set, taking care not to add undue measurement burden.

The challenge of addressing quality is compounded because fragile, conflict-affected and vulnerable settings do not represent a homogenous set of circumstances, but rather a series of unique settings. Services provided may be directly supported or sometimes provided by humanitarian and development agencies. Over the past 20 years, the international humanitarian community acknowledged the need to ensure standards, such as through the Sphere project.^11^ The formulation of the Core Humanitarian Standard on Quality and Accountability, technical guidelines and indicators adapted to such settings have all been proposed. Many of these initiatives address some of the eight elements mentioned above. Building on this foundation, the global health and humanitarian community should meet the challenge of delivering quality services that are effective, safe and people-centred. 
